# What is it like to be the Metaphysical Subject? An Essay on Early Wittgenstein, our Epistemic Position, and Beyond

**DOI:** 10.1007/s11406-016-9693-z

**Published:** 2016-02-09

**Authors:** Konrad Werner

**Affiliations:** Institute of Philosophy, Jagiellonian University in Krakow, Grodzka 52, 31-044 Krakow, Poland

**Keywords:** Wittgenstein, Metaphysical subject, Epistemic position, Qualia, What is it like, Resolute reading

## Abstract

I argue that Ludwig Wittgenstein’s idea of the metaphysical subject sheds new light on subjective qualities of experience. In this article I draw first of all on the interpretations provided by Michael Kremer and James Conant. Subsequently, I conclude that “what is it like” means primarily “what is it like to see myself as the metaphysical subject”.

## Introduction

To begin, I shall bring up the following lines from Ludwig Wittgenstein’s *Tractatus logico*-*philosophicus*. However, let me try to read these words in a somewhat unusual way, and indeed to take them at their face value: as first-person statements by a man reflecting on his life:

5.6 The limits of my language mean the limits of my world.

(…)

5.62 (…)

That the world is my world, shows itself in the fact that the limits of the language (the language which only I understand) mean the limits of my world.

5.621 The world and life are one.

5.63 I am my world. (The microcosm.)

(…)

5.641 (…)

The I occurs in philosophy through the fact that the “world is my world”.

I feel emboldened in this reflexive reading of Wittgenstein’s *Tractatus* by the ingenious and gripping interpretation provided by Michael Kremer (2001, 2004) and by Wittgenstein himself, who clearly stated in the letter to his publisher that the point of the book was ethical (see Wittgenstein 1971). I believe that these fragments, among others, express what I have elsewhere called a recognition of *epistemic position* (see Werner, submitted for publication); that is to say, of the limits of knowledge ensuing from the fact that we are always presented with some aspect, side or mode of presentation of the world; from the fact that the world *is* a certain way *for* us, as Crane (2001) aptly puts it.

I would like to situate Wittgenstein’s conception of the metaphysical subject within this frame of reference. Although this article cannot be considered as belonging to Wittgensteinian scholarship in the strict sense, I nonetheless argue that the appeal to epistemic position yields gains in the interpretative task as well by providing linkage between the metaphysical subject, which is often seen as being an empty, tautological idea, and the empirical, psychological subject, that is the one who undertakes philosophical reflection. Moreover, I am going to show that taking these two ideas together we can tackle the problem of subjectivity – the “what is it like” phenomenon – anew. I conclude that even if there is no special experiential “what is it like” e.g. when I taste my morning coffee, nonetheless there may still come up a special “what is it like” accompanying *my* recognition of *my epistemic position*. In other words, the recognition itself is subjectively *experienced*.

This essay may then appear a piecemeal work, torn apart by several philosophically pregnant topics, too demanding to be addressed in one relatively short exposition. Even more so when I bring up “what is it like” which seemingly has nothing to do with Wittgenstein’s early period (Block 2007 addresses Wittgenstein’s writings from the early 1930s and refers to qualia, but here my focus is exclusively on the *Tractatus*). However, establishing the links between these blocks is precisely my purpose. Here is an overview:

First, I shall introduce the notion of epistemic position; second, I will recognize Wittgenstein in this light, drawing mostly on the insights of Kremer (2001, 2004), and James Conant (2000, 2002). When highlighting the connection between Wittgenstein’s idea of the metaphysical subject and the idea of epistemic position, the latter is, I believe, accurately accounted for, while the former can no longer be regarded merely as a tautological construct purported to upend all philosophical positions and curb all philosophical efforts. Finally, drawing on this interpretation of Wittgenstein, I shall address the problem of the “what is it like” quality of experience.

## Epistemic Position

For now, in order to start without a bigger philosophical baggage, let me suppose the usual, “folk” and hardly definable understanding of the word “world”. The world is what I have around me and what you have around you; this is what we have *in view*, so to speak. The notion is thus initially as primitive as it could be, and it may be provided with a more or less sophisticated philosophical refinement in the subsequent reflection, however, none of such refinements should be prejudged in the first step.

Now, the recognition of one’s epistemic position is precisely *about this familiar world* in which we live, and interestingly enough, each step of it seems obvious *after* it has been completed. After all, could there be more obvious claim than Wittgenstein’s recollection that (5.641) “world is my world”, not to mention Descartes’ recognition “Ego cogito, Ego sum”. Indeed, 5.641 and some of the other seemingly obvious theses of the *Tractatus* are often deemed tautologies lacking any content whatsoever (see Hintikka 1958; Lugg 2013).

According to my overview, epistemic position of the subject is constituted by at least the following four factors (this list is by no means exhaustive):

(1) Firstly, by the subject’s *location*.

Hilary Putnam in known, among many other things, for his rejection of the “God-Eye View”, which has prevailed, as he holds, in modern epistemology. This means the “epistemic ideal of achieving a view from an ‘Archimedean point’ – a point from which we can survey observers as if they were not *ourselves*, survey them as if we were, so to speak, *outside our own skins* (…)” (Putnam 1990: 17). Henry Allison calls this prevailing epistemological view a theocentric epistemology, since it seems appropriate for God, if any, but surely not for the *finite* minds. According to this view “human knowledge is to be measured and evaluated in terms of its conformity (or lack thereof) to the norm of a putatively perfect divine knowledge” (Allison 2006: 114).^1^ Recently, in the same spirit, Gasparyan (2015) has discussed and criticized the idea of a Global Observer, who is supposed to be capable of gaining a View from Nowhere, as Nagel (1986) would say.

In opposition the “God-Eye View” and theocentric ideal of a View from Nowhere, *being in epistemic position* means in the first place being *somewhere*, being in a particular *position* with respect to all other beings and thus having local perspective(s); it means living inside the world and dealing with it from the inside; it means being saddled with all limitations imposed by the very fact that we are, as subjects, *positioned*.

(2) Secondly, by the subject’s *perceptual capacities*.

Philosophers since the time of Heraclitus have been aware of the fact that our senses provide us only with fractional data, therefore it seems reasonable to distinguish between the world as it allegedly is *in itself* and the ways in which it appears to us via perception. Early modern theories of perception, based on Galileo’s mathematical physics and Kepler’s optics, created a gulf between mechanistically thought of *external* reality on the one hand, and sensible qualities of the world as it is known to perceivers on the other (see Ben-Zeev 1984; Osler 2008). On this basis Rene Descartes and John Locke laid down the categorical scaffolding of primary and secondary qualities which - under different descriptions - would later become a standard also for empirical studies, and it still prevails in cognitive science (for the critique of primary-secondary distinction see Putnam 1987).^2^ The distinction has been recently revamped in the philosophy of perception by Shoemaker (2006); Schellenberg (2008), and Genone (2014), among others (I tackle this problem in Werner 2015).

*Being in epistemic position* thus means that local subjects face an *appearance* of the world that fits with their perceptual capacities. The appearance of the world that I know is determined by the capacities of my receptors, by the whole machinery of my neural network, by the bottom-up mechanisms of selection, etc.

(3) Thirdly, besides perceptual constraints which we share with the rest of animal kingdom, *human* epistemic position is constituted also by *intellectual* machinery of language and rational thinking.

One might argue that the human mind transcends the limits imposed by biological factors, and admittedly that might be the case; this, however, does not change the seemingly obvious fact which I am highlighting in this section, namely that our worldview is still *determined* by these intellectual conditions. Whether or not our intellectual virtues broaden the range of properties of the world available to us, my point is that they are *among the factors* that determine how the world *appears* to us instead of conveying a glimpse of an alleged reality, disregarding or circumventing appearances. John Searle says in this context that “all intentionality is aspectual. Seeing an object from a point of view, for example, is seeing it under certain aspects and not others. In this sense, all seeing is ‘seeing as’. And what goes for seeing goes for all forms of intentionality, conscious and unconscious” (Searle 1992: 131). We are thus accustomed to impose categorical architecture on the world so that we see objects and properties, processes and events on the timescale; we see relations, causes and effects, etc.^3^ Putnam writes:

(…) what is by commonsense standards the same situation can be described in many different ways, depending on how we use the words. The situation does not itself legislate how words like “object,” “entity,” and “exist” must be used. What is wrong with the notion of objects existing “independently” of conceptual schemes is that there are no standards for the use of even the logical notions apart from conceptual choices (Putnam 1988: 114)

Hence, *being in epistemic position* means for humans that they organize their worlds by imposing categorical and conceptual frameworks on them.

(4) Finally, the subject who recognizes her local position along with all other constraints is the one who says “I,” and this very expression opens up a new dimension of epistemic position: I recognize this seemingly obvious “fact” that all facts are *for me*.

Recently Dan Zahavi and Uriah Kriegel have aptly called this feature “for-me-ness”, combining it with “what is it like” phenomenon. They write:

Arguably, for every possible experience we have, each of us can say: whatever it is like for me to have the experience, it is *for me* that it is like that to have it. What-it-is-like-ness is properly speaking what-it-is-like-*for*-*me*-ness. (Zahavi and Kriegel 2016: 36)

Our view is not that in addition to the objects in one’s experiential field – the books, computer screen, half empty cup of coffee, and so on – there is also *a self*-*object*. Rather the point is that each of these objects, when experienced, is given to one in a distinctly first-personal way. (Ibid. 38)

Thus “the world is my world” (5.641), and at the end of the day, if Descartes is right, this is the only thing that I can be sure of. Thus *being in epistemic position* means that the world *I am in* is always the world *for me*. I am a constant and necessary point of reference, as it were, for whatever I could call *a world*.

To sum up, “epistemic position” functions in the beginning as an umbrella term subsuming these differing, albeit correlated constraints imposed on my/our *knowledge*, and, as a matter of fact, on my/our *being in* the world. The list (1) - (4) can by no means be regarded as novel since all these things are well known. The label “epistemic position” is not intended here to merely highlight the fact that there *are* cognitive constraints imposed on our worldviews - this fact is widely recognized, intensively investigated and, in a sense, familiarized in philosophy; it is rather intended to stress the fact that these constraints are something that each of us can *recognize* and that this is a pregnant recognition: it may be *experienced* by me in some way, and as such it can yield a re-recognition of myself *and* the world, having ethical and metaphilosophical consequences.

The philosophy of epistemic position,^4^ conceived as an integrative project, seeks in the first place to assort the variety of issues elicited by (1) - (4) in such a way as to make them eligible for *integrated* philosophical reflection: the kind of reflection that was undertaken by Kant and Husserl, among many others. And in this light I recognize Wittgenstein’s life-long (and changing throughout his life) investigations.

## Setting Wittgenstein in the Context

In the next two sections I’m going to show that Wittgenstein recognizes his epistemic position in the *Tractatus*, and, by saying that the book “will perhaps only be understood by those who have themselves already thought the thoughts which are expressed in it—or similar thoughts” (Wittgenstein 1922: 23), he invites us to follow him (I draw this *engaged* reading mostly on Diamond 2000 and Conant 2002). First, however, let me set up the discussion. Someone might arguably doubt whether there is any clear connection between the early Wittgenstein and the problem of epistemic position as expressed above. Therefore, the first thing to do is to elicit this connection. I will attempt to do this by bringing up the ongoing controversy over Wittgenstein’s understanding of his own work and of the whole philosophical enterprise.

According to the *Tractatus*, the world is a realm of facts (“what is the case”), not objects (1, 1.1); facts are structured complexes of objects (2.01, 2.0272, 2.031, 2.032). Objects are simples (2.01) and they are the substance of the world (2.021). When I know an object, I know all possible complexes that it can form (2.0123). Moreover, if the world is a realm of facts but not objects, and knowledge is knowledge *of* the world, not of anything else, then to know an object *means no more than* to know the possible and actual facts that an object is determined to compose. Hence, substance is apparent and accessible to subjects only via complexes (facts). This claim is further supported by Wittgenstein’s theses that explicitly restrict the accessibility of the world to the limits of language (5.6) and individual life (5.621, 5.63).

Knowledge consists of pictures of facts (2.1). Thoughts are logical pictures of facts (3), whereas a proposition is the perceivable expression of a thought (3.1). What the picture represents is its sense (2.221). A proposition is true in virtue of the correspondence between its sense and the world (2.222). Science is the totality of true propositions (4.11). What then is philosophy? Wittgenstein writes:

4.111 Philosophy is not one of the natural sciences. (The word “philosophy” must mean something which stands above or below, but not beside the natural sciences.)

4.112 The object of philosophy is the logical clarification of thoughts.

Philosophy is not a theory but an activity.

A philosophical work consists essentially of elucidations.

The result of philosophy is not a number of “philosophical propositions”, but to make propositions clear.

Philosophy should make clear and delimit sharply the thoughts which otherwise are, as it were, opaque and blurred (Wittgenstein 1922: 44).

According to 4.112, a philosopher is unable to ask or answer any question posed *within* the domain of facts, so to speak; that is to say he is unable to refer to this or that particular fact. On the other hand it seems that there is still something for a philosopher to do, generally speaking, *on the side of facts*. But what? In the closing remarks of his book Wittgenstein writes:

6.54 My propositions are elucidatory in this way: he who understands me finally recognizes them as senseless, when he has climbed out through them, on them, over them. (He must so to speak throw away the ladder, after he has climbed up on it.)

He must surmount these propositions; then he sees the world rightly (Wittgenstein 1922: 90).

Hence, although philosophy does not produce any theses *on* facts, it is an *activity* striving to *see all facts* anew - to see “the world rightly”.

The best Wittgensteinian scholars have struggled to understand Wittgenstein’s account of philosophy, and consequently, his own intellectual efforts (see e.g. Fogelin 1996; McGuinness 2002; Horowich 2012). Here I would like to tackle a more specific problem: the question of how can we can come to terms with the nonsensical character of the Tractarian theses proclaimed in 6.54, if we keep in mind the provision articulated by Wittgenstein in the Preface, that “the truth of the thoughts communicated here seems to me unassailable and definitive” (Wittgenstein 1922: 24). How can anything be nonsensical, yet still true?

Several strategies have been undertaken, and there is no place to explore the issue here in detail, however, let me highlight some possibilities.

The biggest gulf looms between the so-called New Wittgensteinians and a more traditional approach drawing on the distinction between *speaking* (representing) and *showing*. Wittgenstein writes:

4.12 Propositions can represent the whole reality, but they cannot represent what they must have in common with reality in order to be able to represent it—the logical form.

To be able to represent the logical form, we should have to be able to put ourselves with the propositions outside logic, that is outside the world.

4.121 Propositions cannot represent the logical form: this mirrors itself in the propositions.

That which mirrors itself in language, language cannot represent.

That which expresses itself in language, we cannot express by language.

The propositions show the logical form of reality. They exhibit it.

(…)

4.1212 What can be shown cannot be said.

In accordance to *speaking* - *showing* distinction Elisabeth Anscombe suggests that there are two kinds of nonsense employed in the *Tractatus*. If philosophical theses are nonsensical, and if “an important part is played in the *Tractatus* by the things which, though they cannot be ‘said’, are yet ‘shown’ or ‘displayed’” (Anscombe 1959: 162), then it seems reasonable to conclude that there is a plain or a misleading, thus useless nonsense, a gibberish, *and* there is a “showing”, substantial or an illuminating nonsense - when a speaker does not obey “logical syntax”, nevertheless can deliver a message (see also Hacker 1986). James Conant, who strongly rejects the *two*-*nonsense* view, claims that it ensues from “the doctrine that there are *certain aspects of reality* that cannot be expressed in language but can nonetheless be conveyed through certain sorts of employment of language” (Conant 2000: 178). On the other hand, this conception follows, in a sense, the famous remark made by Bertrand Russell in his *Introduction* to the *Tractatus*, namely that “Mr Wittgenstein manages to say a good deal about what cannot be said” (Russell 1922: 18). As such, the illuminating nonsense - the one being often exercised by philosophers - is valuable as long as it enables us to see what cannot be said, and what turns out to be of the greatest importance to human life (see e.g. 6.41, 6.522).

Let me put these things in a simple, and hopefully not simplistic, way: the idea of illuminating nonsense, as I understand it, attempts to do justice to what I shall provisionally call *a sense of modesty*– quite a commonsensical stance. It points to the fact that (a) we have no reason to believe that our linguistic tools are tuned to express all that we owe, so to speak, to the world – that is to say, all our perceptions, feelings, attitudes. Moreover, (b) we have no reason to believe that what we owe to the world or what we receive from it is everything the world can offer, everything that the world contains. Taking (a) and (b) together, it seems reasonable to suppose that there are *certain aspects of reality*, as Conant puts it with an overt reservation, that cannot be expressed properly in language; we can nonetheless express them by using language much as we use gestures or illustrations: by showing rather than speaking in the strict sense of the word. Arguably the sense of modesty ensues from the recognition of epistemic position as described in (1) - (4).

More or less with conjunction with the traditional view (i.e. the one endorsing the two kinds of nonsense) one can take the route of holding that Tractarian theses are tautologies, thus they are not so much nonsensical, but rather empirically trivial or empty. According to Lugg (2013) they, as tautologies, do not represent facts, nevertheless they show logical structure of the world and in doing so they are much closer to the mathematical truths rather than the empirical ones. However, it is not entirely clear to what extent we can employ this conception of tautology in reading other philosophers, thus to what extent this meta-Wittgensteinian account, so to speak, can serve as a more general metaphilosophical template.

As I have already pointed out the traditional view has been bitterly criticised by the New Wittgensteinians’ movement set in motion by the writings of Cora Diamond (1991) and Conant (2000). According to this reading (called sometimes “resolute reading”) there is just one kind of nonsense - the plain one - and Wittgenstein attempts precisely to demonstrate this fact in the *Tractatus*. If I’m not wrong, we could say that he tries to do this not only *in* the *Tractatus*, but *by* the *Tractatus* itself. Conant (2000) argues that Wittgenstein’s conception of nonsense stems from *his* acknowledgment of the tension occurring in Gottlob Frege’s conception of nonsense, namely from Frege’s hesitation whether to proclaim two kinds of nonsense or just one. Wittgenstein is supposed to have preferred and strongly recommended the latter option, whereas Rudolf Carnap was a proponent of the former and, if I understand Conant correctly, this fact might have misled traditional interpreters of the *Tractatus*.

Conant summarizes his position in the following way:

To recognize a *Satz* as nonsensical [*Unsinn*], for the *Tractatus*, is not a matter of recognizing that it is attempting to say something that cannot be said, but rather a matter of recognizing that it fails to say anything at all. (Conant 2000: 194)

On the Tractarian conception, there is only one way a sentence can be *Unsinn*: by its failing to symbolize. This conception does not rule out the possibility of *Sätze* (such as tautologies and contradictions) which have logical structure and yet are devoid of *Sinn*. (…) It only rules out a sentence’s having an “impossible sense”—a sense that it *cannot* have because of the senses that its parts already have” (Ibid.: 194–5)

Hacker (2003) comes up with a blistering criticism of the New Wittgensteinians arguing that they misrepresent both Wittgenstein and Carnap (see also Proops 2001). I am not going to investigate it, however, I believe that New Wittgensteinians are right at least in one crucial respect: they correctly point out an obscurity in targeting *what cannot be expressed* in language as a quasi-fact. To show this I refer to Anscombe. She holds with respects to these “things” that cannot be said that “it would be right to call them ‘true’ if, *per impossibile*, they could be said”; but actually “they cannot be called true, since they cannot be said, but ‘can be shewn’” (Anscombe 1959: 162). It is not entirely clear what these “things” are, but if we take this statement seriously at face value, then what Anscombe means is that it is *possible* to call these “things” true, but impossible to express them, so their *actual* qualification *as true* cannot be the case. Therefore, the “things” are such that it is *possible* for them to be true and at the same time it is *impossible* for them to expressed. Let me put the emphasis on that point: the lack of *actual expression* stems from the *impossibility of any expression*, but the lack of actual qualification as “true” does *not* stem from the impossibility of such qualification, but merely from the lack of actual expression. At the end of the day we cannot call these “things” true because it is impossible to express them, *not* because the very category of truth makes no sense with respect to them. As long as we endorse the more or less standard view that facts are truth-makers, Anscombe’s claim suggests that what cannot be expressed in language is still *a fact* of some very special sort.

Two things come to mind in this context. First, if the inexpressible “thing” is a fact or quasi-fact, and we actually *do* express many facts, then *why in principle* deny that these special quasi-facts can be expressed? Are there any reasons for that? Secondly, the sense of modesty which I recognized as underpinning the two-nonsense interpretation now works against it: it seems that we have no reason to believe that the template actually used by us to organize the perceived world – the one established inter alia by the category of fact – is *in reality* employed independently of our mastery of language. Must these “things” really be *facts*?

Note what Wittgenstein writes:

5.634 (…) Everything we see could also be otherwise. Everything we can describe at all could also be otherwise (…) (Wittgenstein 1922: 75).

6.4321 The facts all belong only to the task and not to its performance (Wittgenstein 1922: 89).

It is not my goal to get a handle of this diversity of positions. However, provided that the resolute reading promoted by Diamond and Conant is at least a plausible option; hence if it *may* be the case that the *Tactatus* consists of a plain nonsense, then how could we read it as *true*, as showing something, and as *ethically significant*?

I shall focus on the answer that has been put forth by Michael Kremer (2001, 2004), whose reading of Wittgenstein is a rare example of an intellectual journey that is unchained, so to speak, from the urge to address more and more specialized and detailed puzzles. His reflection spans Wittgenstein, Saints Paul, Augustine, and John of the Cross, Tolstoy, and Kierkegaard, resulting in a comprehensive grasp of the *Tractatus* against the background of a broad intellectual history.

This is the point in which the presented interpretation Wittgenstein meets the idea of epistemic position. The abovementined intellectual history, in Kremer’s view, is the history of human attempts to provide knowledge and action, thus *being in the world*, as Heidegger (and Hubert Dreyfus) would say, with a firm ground; with an ultimate justification - with objective values and objective truths that cannot be threaten by any doubt. Thus this is the history of attempts to attain an Archimedean Point or a God-Eye View, as Putnam says, enabling us to see reality outside our limited perspective. Meanwhile, as Kevin Cahill aptly points out, according to Kremer’s Wittgenstein “we are finite creatures who are unable to provide ourselves with the foundations of knowledge and right action.” (Cahill 2004: 47). Kremer, then, provides us with an account of Wittgenstein’s overall philosophical efforts that places them clearly within the context of epistemic position. What I call recognition of epistemic position stands, due to Kremer’s reflection (of course Kremer does not use this notion), at the very heart of Wittgenstein’s understanding of the philosophical enterprise.

In the preface to the *Tractatus* Wittgenstein writes:

The book will, therefore, draw a limit to thinking, or rather—not to thinking, but to the expression of thoughts; for, in order to draw a limit to thinking we should have to be able to think both sides of this limit (we should therefore have to be able to think what cannot be thought).

The limit can, therefore, only be drawn in language and what lies on the other side of the limit will be simply nonsense. (Wittgenstein 1922: 23)

These words fuel Kremer’s interpretation. According to him, “it is a methodical purpose of the *Tractatus* to bring us to see that philosophical theorizing, conceived as offering us a source of grounding or justification for logic, language or life, will, in the end, produce nothing but such nonsense.” (Kremer 2004: 62).

As a matter of fact, according to Kremer, for a subject who struggles to find such ultimate justification this is a struggle to find a firm ground for her very individuality; the struggle to underpin her position in the world or even, we might add in an Allisonian spirit, to elevate it to a God-like stance. Meanwhile, Kremer continues, Wittgenstein finds his thinking aligned rather with the idea of self-abandonment when he writes down in his diary:

I cannot bend the happenings of the world to my will: I am completely powerless (Wittgenstein 1979: 73e).

Hence, Kremer believes that the rejection of “all forms of self-assertion, including the self-assertion found in certain misguided forms of asceticism, piety and false humility, is what the *Tractatus* really aims at”. (Kremer 2004: 60). The *Tractatus* “aims to relieve us of this need for ultimate justification by revealing that all such justificatory talk is in the end meaningless nonsense” (Kremer 2001: 51). The book is directed against the attitude targeted by Allison (2006) as theocentric epistemology: the theocentric ideal for human cognitive capacities. We must therefore abandon the “prideful hope that we can give meaning and value to our lives” (Kremer 2001: 56).

Finally, to account for the truth and ethical significance of the *Tractatus* as conceived by the New Wittgensteinians, Kremer points out how we should understand the two terms propelling the whole discussion, i.e. “truth” and “showing”. When it comes to the latter Kremer urges that we must not think of it as “a *relation* between a subject and some ineffable fact-like entity (…). This form of the idea of showing is exactly what the *Tractatus* wants to teach us to abandon. Rather, we should read talk of ‘showing’, and correlatively ‘seeing’, on the model of the demonstration of a technique” (Kremer 2004: 62). Showing is like “dancing the waltz”, which “should be understood simply as ‘waltzing’ rather than as involving a relation between a dancer and a particular ‘dance’ (the waltz).” (Ibid.). I seems that Anscombe’s and Hacker’s accounts of showing are predominated by Wittgenstein’s idea of picturing facts, despite his explicit declarations that showing is precisely opposed to picturing. Hence, Tractarian showing is not a special picturing but rather exercising “the practical abilities and masteries that are part of our ongoing talking, thinking and living.” (Ibid.: 63). Therefore “to show” in the Tractarian sense is not like “to show a picture” which is a relation between two persons and a picture, but rather it is like “to show how to dance” which is a relation between two persons or, as a matter of fact, between their actions.

Tractarian “truth” is in Kremer’s reading also, so to speak, *enacted*:

There is another sense of ‘truth’ which we can appeal to here, exhibited in such Biblical passages as: ‘…whoever does the truth comes into the light… .’. ‘Truth’ as something which one can *do* is not something we might be tempted to think of as expressible in a proposition. It is, rather, a way to be followed, a ‘path’ for life. Insofar as the *Tractatus* communicates a ‘truth’ it is by demonstrating to us, in practice, how to follow such a path” (Kremer 2004: 63)

Hence, the *ethical truth* that we are invited to discover in the *Tractatus*, is not a nonsensical proposition purported to maintain some special picturing of some special ineffable facts but a path of life and thinking, *exercised* by Wittgenstein *in* the *Tractatus*; the one that should be undertaken by us as well. This truth thus cannot be *conveyed by* any propositional deliberation; it is showed over the course of *exercising* plainly nonsensical deliberation attempting to find a firm, ultimate metaphysical grounds for the world, for the subject and for knowledge. The crucial thing to be acknowledged is that, as readers, we are necessarily *engaged in nonsense*. Conant puts it explicitly by saying that “we are drawn into the illusion of occupying a certain sort of a perspective. (…) from which we can view the logical structure of language ‘from sideways on’ (…) The assumption underlying Tractarian elucidation is that the only way to free oneself from such illusions is to fully enter them and explore them from the inside (…)” (Conant 2002: 422).

To sum up, the acknowledgement of our locality and finiteness, of our inability to attain a God’s Eye perspective and ultimate foundations, is a key part of what I mean by the recognition of epistemic position.

## Wittgenstein on the Metaphysical Subject

In this section, I shall reconstruct the Tractarian conception of the metaphysical subject and so-called solipsism. In my reading, this is the core of Wittgenstein’s recognition of epistemic position. From this standpoint, although Conant’s and Kremer’s views served as templates for my exposition of the connection between Wittgenstein and the idea of epistemic position, I shall be able to show the limitations of resolute reading.

Wittgenstein starts his path to the recognition of epistemic position by identifying the limits of what can be cognitively accessed with the limits of what can be said:

5.6 The limits of my language mean the limits of my world (Wittgenstein 1922: 74)

We must remember that for Wittgenstein at the time of the *Tractatus* (as for Frege) “language” does not mean English or Chinese, but universal language, as Putnam puts it, “of which all the natural languages are different realizations. That is, there is a fixed totality of possibilities, or, as one might also put it, of coherent thoughts to think, which are simply expressed in different signs in different natural languages” (Putnam 2008: 5). However, the subsequent theses provide further relativisation, yet not only to language, but rather in a spirit closer that of the *Philosophical Investigations* (Wittgenstein 1953) to life.

5.621 The world and life are one.

5.63 I am my world. (The microcosm.) (Ibid.)

In 5.621 and 5.63 the perspective is more “subjective”. Thus in the *Tractatus* we find a tension between Fregean universality of language on the one hand, and the Schopenhauerian, apparently subjective character of the reference to *my* world and *my life* on the other. I am not going to reconcile these contrary tendencies as this is not the aim of this article. However, all these words make it clear that according to Wittgenstein, we cannot think of the world *of facts* without taking into account the subject.

In this spirit, according to Stenius (1960); Westphal (2005); Tang (2011) and many others, Wittgenstein’s conception appeals to the transcendental idealism of Immanuel Kant, as it “seeks to make manifest the inseparable connection (a) between thought and reality and (b) between mind and world” (Tang 2011: 598). On the other hand, however, Wittgenstein explicitly states that (5.634) “[t]here is no order of things a priori” (Wittgenstein 1922: 75). So, Wittgenstein’s appeals to Kant are mixed with apparently anti-Kantian threads. Moreover, as Kremer convincingly showed, Wittgenstein rejects the idea of subject as “the author, the structurer, the ground of all Being”, as “privileged centre, a fixed point of origin for the coordinates of logical space, as it were” (Kremer 2004: 71). Instead, he strongly endorses a mystically-inspired view of the “empty” subject. He writes in his diary:

The world is *given* me, i.e. my will enters into the world completely from outside as into something that is already there (…)

That is why we have the feeling of being dependent on an alien will.

However this may be, at any rate we are in a certain sense dependent, and what we are dependent on we can call God (Wittgenstein 1979: 74e)

This fragment is crucial if we want to apprehend this *Kantian*-*Anti*-*Kantian* mixture; to apprehend the proper sense of Tractarian transcendentalism; and to understand why, at the end of the day, Wittgenstein’s conception of the subject and its relation to the world turns out to be – as the philosopher himself stresses – realism (5.64). Interestingly enough, this is a realism that binds subject and world together as a non-separable pair. We have then at least two philosophical tensions here: firstly, between mystical abandonment of the subject on the one hand, and making facts, in the transcendental spirit, somehow subordinate to the subject on the other; secondly, between the subject-world bond, again, and proclaimed realism.

The core of these somewhat surprising conclusions is Wittgenstein’s conception of the *philosophical I* or the *metaphysical subject*. His argumentation for that notion starts with the “isolation” of the subject; a method which in some respects runs parallel to that of Descartes’ *Second Meditation*, where in search of indubitable knowledge the self – the thinking substance – is isolated, step by step, from everything external to it; from everything that is not *necessarily* related to it. Kremer observes:

So the author of the *Tractatus*, seems to himself to have followed the path of knowledge to achieve a mastery over language and the world; thus both language and world have become, for him, *mine*. (Kremer 2004: 70)

However, from this perspective, Descartes seems to have stopped half way,^5^ since he seems to remain with two distinct domains *of facts* – external (physical objects) and internal (mental ideas) – whereas Wittgenstein is much more radical: during the course of isolation, the subject *departs* the domain of facts, as it were:

5.631 The thinking, presenting subject; there is no such thing.

If I wrote a book “The world as I found it”, I should also have therein to report on my body and say which members obey my will and which do not, etc. This then would be a method of isolating the subject or rather of showing that in an important sense there is no subject: that is to say, of it alone in this book mention could not be made (Wittgenstein 1922: 74).

5.632 The subject does not belong to the world but it is a limit of the world.

Kremer comments:

Yet this ‘achievement’ of mastery over the world begins to unravel before it is even finished being proclaimed. For 5.61 reminds us that, according to the theory of language as pictorial representation developed in the earlier part of the book, the limits of language and the world are also the limits of *logic* and so of *thought*. Logic, language and thought cannot ‘get outside’ these limits, for that would be, as the Preface warned us, only to produce nonsense. Yet if we can say ‘there is the I’ then this ‘I’ cannot be *in* the world, for ‘we cannot … say in logic: This and this there is in the world, that there is not’. (Kremer 2004: 70)

There is no fact that is *necessarily* related to the subject, because within the domain of facts *nothing is necessary*; everything “could also be otherwise”. As Jakko Hintikka outlines, Wittgenstein “is interested only in what can be said to be mine *necessarily*; for otherwise he would only be doing empirical psychology. But the only necessity there is, according to the other doctrines of the *Tractatus*, is the empty tautological necessity of logic. There is nothing, therefore, in the world which can be said to be mine in the relevant sense of the word” (Hintikka 1958: 89).

On the other hand, however, the whole domain of facts is subject-related. In this context Wittgenstein introduces the idea of a metaphysical subject:

5.641 There is therefore really a sense in which in philosophy we can talk of a non-psychological I.

The I occurs in philosophy through the fact that the “world is my world”.

The philosophical I is not the man, not the human body or the human soul of which psychology treats, but the metaphysical subject, the limit—not a part of the world (Wittgenstein 1922: 75).

Does it mean that the metaphysical subject is by no means related to the psychological (empirical) subject? I will come back to this question later. Meanwhile, the metaphysical subject is characterized as a necessary point of reference, as it were, for the world of facts:

5.633 Where in the world is a metaphysical subject to be noted?

You say that this case is altogether like that of the eye and the field of sight. But you do not really see the eye.

And from nothing in the field of sight can it be concluded that it is seen from an eye (Wittgenstein 1922: 74–75).

Wittgenstein writes that from nothing *in* the field of sight can it be deduced that there is an eye, however let me note that the very notion of the field *of sight* is incomprehensible unless there is an eye *for which* a given field *is* or *serves as* the field *of sight*. The field of sight must be, so to speak, attributed to the eye, otherwise it is not the field *of sight*. Similarly, there is no fact *in* the domain of facts which requires the existence of mind, but the existence of the whole domain is attributed to mind: a given domain cannot be the domain *of facts* unless there is also a cognizing subject *for which* it is the domain of facts. The subject is then conceived of as a limit of the world, not a part of it, like the eye, which is the limit of the field of sight. Being a limit of the domain *x* is here conceived of, if I’m not wrong, as being a necessary condition for the *demarcation* of *x*, which is not itself a concrete element of *x*. In fact, it is a theoretical abstract. Think of any material object, be it for instance a book on your table. As the book is distinct from the table, there must be a limit for the book, but the latter (or the boundary between the book and the table) is not as much a concrete element of the book as sheets of paper are. Limits belong to books in a different sense than do sheets of paper. Analogously, the metaphysical subject belongs to the world in a different sense than facts do. Hence, the subject, conceived of as the limit, is the necessary condition for any *demarcation of facts*; and thus for any *appearance of the world* in the guise of facts. Here Wittgenstein clearly lines up with the transcendental tradition.

At the same time, the subject does *not produce* the world, as the eye obviously does not produce its field of sight; if so, then apparently Wittgenstein’s relativisation of facts to the subject is a kind of realism, though admittedly quite an unusual one. He writes:

5.64 Here we see that solipsism strictly carried out coincides with pure realism. The I in solipsism shrinks to an extensionless point and there remains the reality co-ordinated with it (Wittgenstein 1922: 75).

We have then arrived at Wittgenstein’s approach to the subjectivity of epistemic position: “world” means something to me only if it is “my world.” At the same time I am “not a part of the world but a presupposition of its existence” (Wittgenstein 1979: 79e). The subject and the whole realm of facts are, in a sense, *positioned* with respect to each other, however, “the nature of the subject is completely veiled” (Ibid.).

To sum up, Wittgenstein’s enigmatic style leaves room for indeterminacy and elusiveness, nonetheless it is quite clear to me that his remarks point to what I call epistemic position: the subject *lives in* the world, but as long as (5.621) “[t]he world and life are one” and “[t]he World is *given* me” (Wittgenstein 1979: 74e), it lives within a *presentation* of the world *fitting with* its *life*; in the world that *is* a certain way *for* it.

This is the key moment. The idea of metaphysical subject lines up with the recognition of epistemic position. However, the epistemic position in question is not the epistemic position *of the metaphysical subject*; it is the position *of the empirical*, *psychological self* who is engaged in philosophical reflection – engaged in nonsense, Conant says. Therefore these two things are, in my view, inseparably bound together: “isolation” *of the metaphysical subject* and recognition of the epistemic position *of the empirical subject*. The former makes the latter possible: when one comes up with the idea of a metaphysical subject, one’s recognition of epistemic position is complete. At the same time, the recognition of epistemic position connects the metaphysical subject – which is, in itself, a kind of abstract idea or even theoretical fiction – with life, and, so to speak, provides it, as we shall see, with ethical significance.

Someone might argue that in light of Wittgenstein’s account of language at the time of the *Tractatus*, and his critique of a priori truths, solipsism and the metaphysical subject cannot be given any substantial content but should be understood as indicators of the redundancy of both the solipsistic *and* realistic discourses, and thus of the redundancy of philosophy as such. In these circumstances, as Tang (2011) points out, solipsism is an empty position. Hence, according to this view, in Wittgenstein’s reconciliation of solipsism with realism “the point was to say that the world is my world has as little real content as saying that a thing is identical with itself” (McGuinness 2002: 135); and thus when I say that the world is what I experience, and someone else says that the world is the realm of physical entities independent of my experience, there is no significant difference from the perspective of empirically verifiable propositions of science. Both philosophical stories about the world change nothing as regards scientific knowledge of facts. Wittgenstein’s solipsism thus must not be conceived of as a full-blown philosophical position.

However, there is still the question, of which McGuinness (2002); Putnam (2008) and Tang (2011), all analyzing the Tractarian solipsism, are perfectly aware, of why Wittgenstein introduces “my” in 5.6, 5.63, and 5.641 (see Diamond 2000 for comparison). If we claim, as for example Hintikka does, that the subject is for Wittgenstein identical to universal language, or that the limits of the self are nothing more than the limits of language (see Hintikka 1958: 89), then “my” is empty. That is to say, in “my world” the word “my” does not modify the meaning of “world”. As McGuinness explains, I am a completely *neutral point of view* of the world. Similar position is endorsed by Kremer (inspired by Conant):

[t]he *Tractatus* rejects as illusory the very notion of a ‘limit’ of language or the world; but to do so is to reject as illusory the notion of the ‘metaphysical subject’ which *is* a ‘limit’ of the world (Kremer 2004: 66)

Wittgenstein’s analogy of the ‘I’ of solipsism with the eye of the field of vision can help us to see why it is necessary to destroy this ‘I’ (Ibid.: 77)

On the other hand, however, Wittgenstein notes down in his diary:

I can only make myself independent of the world - and so in a certain sense master it - by renouncing any influence on happenings (Wittgenstein 1979: 73e)

Making himself independent of the world can hardly be thought of as Wittgenstein’s attempt to destroy himself. It seems that Hintikka, McGuinness and others, including Kremer, ignore the fact that Wittgenstein does not merely *target* “I” in his analysis but he carries out this analysis genuinely *from the first*-*person perspective* (on reading the Tractarian first person statements see Diamond 2000; Kállay 2012). Renouncement just mentioned by him is not a destruction of some empty idea; this is a change of personal attitude toward the world undertaken in order to “master” it; that is to say, if I’m not wrong, “to see the world rightly”. If so, and if his thoughts can only be understood by those who have thoughts these thoughts by themselves - as Wittgenstein himself announces in the introduction to the *Tractatus* - then a reader is also invited *to adopt the first*-*person perspective*.

Now, my point is that even if the metaphysical subject *itself* is completely impersonal, it is *I* who have performed the philosophical “isolation” along the lines described in 5.631, in the course of which this metaphysical subject was uncovered *by me* and, in a sense, *in me*. It is *I* who have been engaged in nonsense – as Conant would say. And this impersonal metaphysical subject uncovered *by me* turns out to be *the means to see myself and the world anew*: to recognize *myself* as the limit of the world, and to recognize the world *as limited* by *me*. In other words: to recognize my epistemic position.

Therefore, Wittgenstein’s “my” is not about making the metaphysical subject more concrete, more substantial; it is about the intimate *connection* between this empty idea and the one who puts effort to uncover it over the course of reflection or meditation. The concrete, psychological self recognizes its epistemic position by engaging itself in the (nonsensical) “isolation” of the metaphysical subject. Thus, as I have already mentioned, the recognition of epistemic position is inseparably coupled with the idea of the metaphysical subject, and – this is my interpretative point – the idea of the metaphysical subject must not be thought of in separation from the empirical subject’s striving to uncover its epistemic position.

One thing should be stressed at this point. As I announced before, when the idea of metaphysical subject is taken into consideration, the limitations of resolute reading show up. I decided to base by reading on the interpretation proposed by New Wittgensteinians because of their groundbreaking idea that the truth and ethical significance of the *Tractatus* is embodied and enacted, as it were; that it is, so to speak, truth embedded in life. Tractarian theses are not true by virtue of some ineffable quasi-facts; they become true in someone’s life when they’re exercised. On the other hand, however, against New Wittgensteinians, there is apparently no evidence in the text that Wittgenstein intends to “destroy” the subject, as Kremer suggests, and that the metaphysical subject is debunked as illusion. It seems that Wittgenstein takes the latter seriously and that by bringing it up he seriously takes the transcendental stance. I hold that resolute reading is unable to account for this move.

Let me argue for the latter thesis and shed some light on the intimate connection between the metaphysical subject and the empirical subject’s striving to uncover its epistemic position yet from a different angle. Kremer refers to the fact (noted, not without reservation, by Russell) that Wittgenstein plunged himself during the first world war into mystical writings of Angelus Silesius, St John of the Cross, among others. In their view, “we are to empty ourselves so that God may fill us” (Kremer 2004: 76). Here is Silesius on the mystical detachment of the self:

II, 144 What is detachment?

What is detachment? I say without hypocrisy:

That it is the will of Jesus in your soul. (citation following Kremer 2004: 77)

Apparently these genuinely religious attitudes posit the *emptied* self as a *receiver* of divine *content*, so to speak. However, this idea goes against Kremer’s view that as a result of destroying the self “we are enabled to simply *act* in the world” (Kremer 2004: 78). On the contrary, it seems that, given the mystical setup of the world, we are not only *doers* but also *receivers*. Now, the point is – in my view – that the mystical abandonment of the substantial self undertaken by Wittgenstein does *not* destroy the self, but leaves it as an *empty vessel* eligible to be filled with “the mystical” that “shows itself” (this idea seems closer to the Jewish mysticism). Nonetheless, crucially, a vessel is still *something*; a vessel has shape or rather *it is a shape*; I am tempted to say that vessels have limits, but it would be even more appropriate and indeed more Wittgensteinian to say that *vessels are limits*. Now, although they *do limit* the content that is being fed into them, they at the same time *do not* destroy or misconstrue this content: the cup does not destroy the water (what could this *mean* after all to destroy the water?). Finally, it is clear that (here Anscombe’s view recurs far from her original stance) there are “aspects” of reality that cannot be expressed, thus limited by a vessel. In this context it means that there is no vessel or a collection thereof that might provide *all shapes that the water could adopt*. But on the other hand, these “aspects” surely are not quasi-facts, simply because the notion of *a fact* makes no sense with respect to a supposed realm outside any vessel.

To sum up, in my reading, “isolation” establishes an intimate connection or even a continuum between my subjective experience, my empirical self, and the impersonal, point-like or vessel-like metaphysical subject, so that this *connection*, even more than the metaphysical subject itself, becomes something significant in my life; thus it becomes - as Wittgenstein stressed in his letter - ethically significant. Namely, it leads to the recognition of my epistemic position and this recognition may lead me to “see the world rightly” (6.54). Let me stress this claim: it is not the metaphysical subject itself, which is an abstract and empty idea, but *its recognition* that comes about as a significant event in my life because it allows me to recognize *my* epistemic position in the world, as if I could see myself and my world in the mirror. In other words – employing a different metaphor – it allows me to recognize myself not as a content, but as a vessel limiting any content, and to recognize *the world* precisely as the content *in that vessel*.

Someone might argue that by stressing the connection between the metaphysical subject and the recognition made by empirical subject, I put forth a kind of naturalization of the transcendental perspective. Surely this issue requires a separate investigation. Let me then answer this way: being inspired by resolute reading of Wittgenstein, and at the same time being aware of its limitations, I would argue that from Wittgensteinian standpoint *the transcendental* is not a domain of quasi-facts or quasi-subjects opposed to *the empirical*; I would say that the *recognition* is transcendental. This recognition *qua act of the empirical subject* can be explained, as any other act of this kind, by empirical disciplines, but this explanation does not undermine the act’s achievement: I can now see that I face a presentation of the world which “could also be otherwise” (5.634) and that all empirical explanations, including explanations of this very seeing, take place *inside the presentation*.^6^ Transcendental recognition comes *from inside* the limited world, Wittgenstein would say, but it strives to uncover the limits themselves. Hence, this aiming at the limits of the empirical domain makes the recognition transcendental.

## Subjective Qualities?

In the last sections of this paper I shall elaborate on how recognition of the metaphysical subject and of one’s epistemic position becomes ethically significant. In order to succeed in this task, however, I have to address very briefly a topic that seemingly has nothing to do with the metaphysical subject and with ethics. Meanwhile I hold that this topic provides precisely the link between them.

Now, if the “philosophical I” *cannot* refer to any fact in the world, and thus also to any fact about my body, then one might conclude that Wittgenstein proclaims, on his own special lines, a purely subjective level or aspect of the conscious mind. This is because, to put it simply, if there is a subjective experience of *what it is like* to be a vessel for scientific facts, then this very experience cannot be a scientific fact. It seems then, one might sum up, that Wittgenstein viewed in this way comes up with the idea of “phenomenal consciousness” (Block 1995), i.e. the one which causes the “hard problem of consciousness” (Chalmers 1995, 1997); that he comes up with “qualia”. I generally agree that “what is it like” has something to do with the metaphysical subject and epistemic position, however, there are some tricky issues regarding the idea of factual content and of reducibility.

According to Nagel (1974); Block (1990), David Chalmers (1995, 1997) and many others there is something about experience which cannot be examined and explained objectively, hence scientifically (at least by today’s science) – the subjective quality. For instance one can say much about the sense organs, physiology, etc., but one cannot *know how the morning coffee tastes to me on my porch on Saturday*. If I gather all such subtle moments of my experience, then it would be reasonable to conclude that one cannot *know what is it like to be me*. The “what is it like” phrase coined by Nagel (1974) is surely the most ingenious way of expressing this peculiar “fact” about consciousness.

However, my use of quotation marks above is not coincidental. The controversy has to do with the possible occurrence of “what is it like” *as a fact*, a component of the domain of facts, successfully explored by the empirical sciences. Qualia or “what is it like” taken in this way – *as facts among other facts* – are stumbling blocks for naturalistic accounts of mind. That is why naturalists attempt to reduce qualia or simply claim that there are no such facts. Friends of qualia are forced into endless withdrawal, in order to distill *still the purer and purer subjectivity* – basic, objectively inexplicable, but still *factual*.

In the Wittgensteinian perspective reductionists are right in one respect: “what is it like” *is not* a fact^7^ about psychological I, pointed out by a proposition obeying the rules of logical syntax. However, as we have just learned from Wittgenstein, there is also a way of speaking that might be treated as a ladder (6.54), and (5.641) “[t]here is (…) really a sense in which in philosophy we can talk of a non-psychological *I*” (Wittgenstein 1922: 74). So, against reductionists, from the Wittgensteinian perspective there is *something* (but *not* a fact) that the “what is it like” phrase *expresses* or *shows*. Now, is it possible to reintroduce “what is it like” in this new context? I shall formulate the proposal of such a reintroduction. “What is it like” is usually a part of apparently non-transcendental considerations in the philosophy of mind; meanwhile together with Rowlands (2003) I argue that this is the crucial misunderstanding: the right place for “what is it like” and qualia is the transcendental stance. I take the latter, however, in the Tractarian sense.

## “What it is like” is a Heuristic Device

I’m getting closer to my final posit. Now, I argue that if the recognition of the metaphysical subject and of epistemic position is to convey any ethical message, there is no reason to believe that only professional philosophers can achieve this discovery; it seems reasonable to suppose that folks can also succeed in this task, even if they lack clarity, deepness or some other virtues (*if any*) that we expect from professionals. Moreover, one could even hold, not without justification, that this professional reflection should not be separated from the folk one.

If so, then there must be a way to make the recognition without plunging oneself into a specialized philosophical analysis like the one conducted by Wittgenstein and like those conducted by Descartes, Kant and Husserl, among many others. There must be, so to speak, a shortcut, a kind of a *heuristic* that might be adopted by anyone. So, here is my final posit: “what is it like” is exactly the philosophical heuristic facilitating the recognition in question.

I have to note that this is the point in which I no longer rely on Wittgenstein or Wittgensteinian scholarship. The idea of a heuristic is crucial here and I shall introduce it is a few words.

When a subject attempts to make a decision in the specific circumstances, she can apply two strategies: first, she could list all possible options, and list all expectations, and finally match the predictable consequences of all the options with the expectations. On this basis, as the result of a detailed computation, the optimal decision can be made. However, in real life, the subject mostly needs to simplify this process by means of a heuristic. The latter is usually conceived of as a shortcut, a simple rule of reasoning, which is not infallible, but sufficient in a particular context (or environment) to provide a solution to the problem or, more generally, to achieve any kind of goal that acting or thinking may have. Heuristics are also useful in learning (see Gigerenzer 2008). Here of course this term is provided with a more relaxed sense. In other words, by philosophical heuristic I mean a relatively simple way to learn or to see a given complex philosophical problem.

Now, the recognition of epistemic position can also be regarded as heuristically-driven learning, however outrageous. Thus, together with philosophical analysis in the strict sense, which might be compared to full-blown problem-solving, there must be a short-cut – a heuristic device that facilitates the recognition. Here is the example; the philosophical literature on qualia is full of them: Imagine that you have to explain *what it is like to be* a passenger on an aircraft to someone who has only seen planes in films, but perfectly understands, maybe even better than you do, all the physical processes which enable the vehicle to fly (this example is in the spirit of Frank Jackson 1986, however, it does not have as much philosophical “baggage” as colour perception). Imagine that you have said everything that can be said about the experiences you have had as an aircraft passenger, and you still *feel* that you *should* say something more, that there is something more in this experience. This “something” is the “fact” that it was *you* who had those experiences. You cannot, for instance, transfer to someone else the very “fact” that it was *you* who experienced the flight. This is because, Wittgenstein would probably say, this “fact” is not a fact; it is the limit of all facts constituting *your* world – the only world you can ever know.

Hence, in my view, “what is it like” is *not a description of a fact*, but a heuristic device that facilitates the recognition of epistemic position, and consequently - of the metaphysical subject. “What is it like” is a heuristic device as long as it enables me to recognise myself anew: as the one *for whom* the world *is a certain way*.

Note that from this perspective it is not so important whether there *are* two kinds of nonsense; that is to say whether a given expression *is* plainly nonsensical or, say, philosophically nonsensical. Rather the question is whether a given nonsensical expression - like the “what is it like” ones - can *serve as* philosophically significant heuristic devices. Therefore, maybe the point in the traditional view is that some of the nonsensical expressions used by philosophers can *serve as* heuristic devices enabling me to see (thus, is a sense, *showing* me) something from the very inside of “my world”, without an ambition to break the limits of thought.

## Qualia

Until now I have used “what is it like” and “qualia” interchangeably. From this point I would like to differentiate these terms: “what is it like” refers to the heuristics discussed above, and by “qualia” I shall mean a subjective quality accompanying the recognition of epistemic position and metaphysical subject. However, somewhat surprisingly, this is a quality experienced by the empirical subject, and as such - it likely is reducible.

Even when I am performing a recognition of the metaphysical subject, however unusual, I am nevertheless empirical, concrete subject, a compound of body and psyche. In other words, “isolation” of the vessel from its content is the *task*, but its *performance* is done by the empirical self located in the physical space and harbouring the whole variety of experiences. And possibly this very recognition, conceived of as a mental act, is accompanied by some special feeling. Think of Descartes for a moment. By employing the overtly *meditational* way of conducting his philosophical analysis - which is not so clear in Wittgenstein’s case - Descartes candidly expresses and shares with us his doubts, hesitations and fears accompanying this intellectual activity.

I would like to put forth the idea that if the recognition of epistemic position *undertaken by* the *empirical* subject and *resulting in* the “isolation” of the *metaphysical* subject is accompanied by some special experience or feeling, then it is precisely *this feeling* we refer to when we speak of *qualia*. Suppose that my experience of drinking my morning coffee on my porch leads, via the “what is it like” heuristic, to the recognition of the philosophical I; e.g. I conduct free-thought experiments while drinking, and as result, I am struck by the “fact” that the whole world I know, including this coffee, is the *world for me*; the world that “tastes” in a certain way *to me*. If there are any specific qualities to *this* recognition, i.e. qualities that no other mental acts have (therefore I do not mean feelings such as pleasure or tiredness, maybe some unique combination of them), then I call them qualia. However, they are *not* the qualities of *experiencing the taste of coffee*, but the qualities of *experiencing the recognition* performed while drinking the coffee. In other words, suppose that the usual, empirically accessible quality of coffee having a certain taste “triggers” (no matter how, e.g. via free association) thought experiments, philosophical analysis and/or the “what is it like” heuristic, then the latter “triggers” recognition of the epistemic position; thus - of the world as *my* world. Now, this recognition, *qua experience*, i.e. as something that happens to me, probably has certain special qualities. These qualities are qualia. Thus they *are* facts, *if* other qualities of experiences are facts, but of no special type. If they are special at all, it is only by virtue of the content of the experience they accompany – it is the recognition which is special, not the manner in which I feel it. *As such*, they are not very interesting to philosophers, and the extent to which they can be explained in a naturalistic manner is then more of an empirical question.

However, even if qualia are not philosophically interesting *qua qualities of experience*, reducible or irreducible, i.e. as feelings “triggered” by the recognition of epistemic position, they nevertheless can be interesting as long as they also “trigger” something more.

Let me recall one of the “ethical” theses proposed by Wittgenstein:

6.43 If good or bad willing changes the world, it can only change the limits of the world, not the facts; not the things that can be expressed in language (…).

In brief, the world must thereby become quite another. It must so to speak wax or wane as a whole (…) (Wittgenstein 1922: 88).

To change the limits of the world means to change the philosophical *I* (as the philosophical I *is* the limit, according to 5.641). As a result of this change, the way the world *is* as a whole also changes; the world “wanes” and then appears anew. Now, probably the most important change that can happen to the philosophical *I* is precisely its self-recognition. I recognise myself as the one for whom the world is a certain way. Note the potential power of this act.

Hence, if my recognition of *my* philosophical *I* – in other words, recognition of *me as a vessel* – allows me to see myself anew, and if it allows me to see the world anew, namely as the world for me, then I can have the *will*, for instance, to hold on to this recognition, to make this change not merely a “flash”, but a real, long-lasting metamorphosis of my attitudes toward my empirical *I* and my surroundings. Thus, toward my life and its environment. As Kremer aptly pointed out:

Solipsism is exercise aimed at bringing about a change in one’s spiritual life (Kremer 2004: 59)

If the special feelings accompanying the recognition of epistemic position and the metaphysical subject, i.e. qualia, “trigger” my will in such way, then they make *ethical* sense. They can modulate my behaviour and the rules I obey. In fact, from Wittgensteinian standpoint, this recognition is a necessary condition of ethical change. This is because:

6.41 The sense of the world must lie outside the world. In the world everything is as it is and happens as it does happen. In it there is no value—and if there were, it would be of no value.

If there is a value which is of value, it must lie outside all happening and being-so. For all happening and being-so is accidental.

What makes it non-accidental cannot lie in the world, for otherwise this would again be accidental.

It must lie outside the world.

6.42 Hence also there can be no ethical propositions.

Propositions cannot express anything higher.

6.421 It is clear that ethics cannot be expressed.

Ethics are transcendental (Wittgenstein 1922: 87–88).

Ethics are transcendental, and so any ethical change in one’s life is, in a sense, transcendental, i.e. it does not refer to special facts (since values are not facts), but to the limits of the whole domain of facts, to what makes them *facts in one*’*s life*. However, on the other hand, there must be something, an impulse inside one’s world, which forces one to make the world appear anew.

Let me put it in the following way. It seems that the recognition of epistemic position, achieved by virtue of philosophical analysis and/or the “what is it like” heuristic, does not necessarily have a stable impact on someone; as I said above – it seems that it can be just a “flash”. Moreover, *as recognition*, it is an intellectual action which does not necessarily have to be (even if usually it is) accompanied by emotions. If so, something else must occur, which forces one to *do* something with this intellectual achievement. And these emotions, experiences or private feelings, could be called qualia. Thus, I would like to propose a redefinition of qualia, or rather their relocation to a new context, so that they become of great ethical significance. They are subjective (reducible or not – it is not entirely relevant here) experiences accompanying my recognition of myself as the one for whom the world is a certain way and of the world as the world for me.

## Conclusion

By the recognition of one’s epistemic position I mean the awareness of limits of knowledge ensuing from the fact that one is always presented with some aspect, side or mode of presentation of the world; the world in which I live *is* a certain way *for* me. I tried to situate Wittgenstein’s conception of the metaphysical subject within this frame of reference, relying partially on James Conant’s and Michael Kremer’s interpretations.

From the standpoint of Wittgensteinian scholarship the appeal to epistemic position yields gains in the interpretative task by providing linkage between the metaphysical subject, which is often seen as being an empty, tautological idea, and the empirical, psychological subject. We are thus able to understand why Wittgenstein comes up with this idea. Moreover, on this basis, I believe, we are able to apprehend the transcendental character of the *Tractatus*, which seems difficult from Conant’s and Kremer’s perspectives.

From the broader standpoint of the philosophy of mind, by taking the idea of recognition of epistemic position and the idea of metaphysical subject together we can tackle the problem of subjectivity – the “what is it like” phenomenon. “What is it like” is a heuristic device that facilitates the recognition of epistemic position, and consequently - of the metaphysical subject. Finally, if the recognition undertaken by the empirical subject is accompanied by some special experience, then it is precisely this experience we refer to when we speak of qualia.

Coming back to Wittgenstein, and taking all of the abovementioned points together we are in a good position to apprehend the ethical significance of the recognition in question in general, and of Wittgenstein’s *Tractatus* in particular. If my recognition of my epistemic position allows me to see myself anew, and if it allows me to see the world anew, namely as the world *for me*, then I can have the will to make this change a real, long-lasting metamorphosis of my attitudes toward my life and its environment. If qualia, i.e. these special feelings accompanying the recognition of epistemic position - accompanying what Wittgenstein calls “isolation” of the metaphysical subject - “trigger” my will in such way, then they make ethical sense.

## Footnotes

1 Allison brings up this critique in his interpretation of Immanuel Kant’s *Critique of Pure Reason*. According to Allison’s reading, Kant is the first modern thinker who rejected the theocentric ideal of knowledgde.

2 More or less independently of these contexts, von Uexküll (1926) introduced the notion of *Umwelt*, that is a species-specific unfolding of the environment, determined by the cognitive tools of a given animal. It is very likely that bats, flies or even dogs, which are relatively close to us, yet equipped with a capacity of smell that is much more sophisticated than ours, unfold different perceptual properties of the world than we do. And, indeed, there is no a priori reason to believe that our worldview is better or more adequate than theirs. Uexküll’s insights line up to some extent with the ideas of Konrad Lorenz, the founder of ethology. Moreover, it seems that similar way of thinking would later be revamped by biological constructivists such as Humberto Maturana and Francisco Varela.

3 Needless to say, these perceptual and intellectual constraints have established what is probably the most intensively explored field in modern philosophy, starting from Immanuel Kant; including Edmund Husserl, Maurice Merleau-Ponty, Martin Heidegger, among others on the one side, and Ludwig Wittgenstein, Kazimierz Ajdukiewicz, Rudolf Carnap, Ludwik Fleck, Karl R. Popper, Willard van Orman Quine, Thomas Kuhn, Donald Davidson, Nelson Goodman, Hilary Putnam, among many others on the other side.

4 What I call epistemic position may also be targeted as an *existential position* if someone prefers to approach the topic from a different - maybe inspired by Kierkegaard, Heidegger or Jaspers - angle. My approach, although it goes beyond the limits of analytic philosophy, keeps maintaining the links with analytic tradition, broadly construed.

5 Someone might argue that this parallel is misconstrued due to the fact that Wittgenstein does belong to the transcendental tradition - on his own special terms - whereas Descartes does not. The latter is true, however I would argue that Descartes paves the way for transcendental argumentation: if I succeed in isolating myself from all contingent circumstances (suppose for a moment that it is actually feasible), I finally find myself outside of the contingent world I know - outside of the domain of facts. *I* am not - when I’m conceived of as a purified, thinking substance - a *fact among facts* and a *thing among things* any more. By stopping “half way” I mean precisely the way to recognition that due to isolation the subject is repositioned, as it were, with respect to the world so that *being a subject* means in the first place *being isolated from the world*; and *being a world* means in the first place *being a point of reference for the isolated subject*.

6 Carr (1999) calls this situation “the paradox of subjectivity”: I can describe myself as an object among other objects in the world, and I can describe myself as a condition of possibility of any objects. This is not the place to discuss this issue, especially whether the term “paradox” is appropriate in this context, but it is surely true that there is a constant tension between these two perspectives. See my [..] for comparison.

7 The peculiarity of thinking of qualia as facts has been convincingly demonstrated by Daniel Dennett (1988). Let me generalize his argument on the Wittgensteinian basis. Facts by definition are complexes (2.01). Hence, if qualia are facts, then they must be complex. If I have complex qualia, then this means that my subjective, privileged and complete knowledge of them - knowledge that is supposed by those who proclaim qualia - requires *in principle* the knowledge of all relations between the simples within them, for instance the relation between tastes and standards of taste as in the case of Dennett’s (1988) hesitant coffee-testers. This means that even if I don’t have this knowledge at a given moment, I *could* have it. Dennett, however, describes a counterexample: the situation where there is no subjective knowledge of such simples. If the coffee which I enjoyed years ago does not taste as fine today, I may not possess the *purely subjective criteria* allowing me to determine whether this taste has changed over the years, or whether my own standards have changed, e.g. they have become more demanding and sophisticated. It is even possible that some empirical, third-personal tests could uncover what has actually happen to my taste. Therefore, there *not necessarily* is a privileged, subjective knowledge. *In principle*, I know no more about what is it like to me to drink coffee than you could know. If so, if in principle there is no special subjective knowledge, there is *no need* to proclaim the existence of any special objects appropriate for that subjective knowledge.
